# 7-Fluoro-6-nitro­quinazolin-4(3*H*)-one

**DOI:** 10.1107/S1600536809046984

**Published:** 2009-11-14

**Authors:** Yundeng Wu, Ancheng Ji, Aihua Zhang, Yipeng Shen

**Affiliations:** a School of Pharmaceutical Sciences, Nanjing University of Technology, No. 5 Xinmofan Road, Nanjing 210009, People’s Republic of China; b Department of Medicinal Chemistry, Jiangsu Provincial Institute of Materia Medica, Nanjing University of Technology, No. 26 Majia Street, Nanjing 210009, People’s Republic of China

## Abstract

The quinazolinone unit of the title compound, C_8_H_4_FN_3_O_3_, is essentially planar, with a maximum deviation of 0.0538 (14) Å for the O atom. The nitro group is twisted by 12.0 (3)° from the mean plane of the quinazolinone ring system. The crystal structure is stabilized by inter­molecular N—H⋯O, C—H⋯N and C—H⋯O hydrogen bonds.

## Related literature

The title compound is used as an inter­mediate for the production of several multi-targeted Raf kinase inhibitors, such as 4(3*H*)-quinazolinone and its derivatives, see: Bridges *et al.* (1996 ▶); Kim *et al.* (2008 ▶). For the anti­tumor activities of quinolines, see: Labuda *et al.* (2009 ▶). For synthetic aspects, see: Rewcastle *et al.* (1996 ▶). For bond-length data, see: Allen *et al.* (1987 ▶).
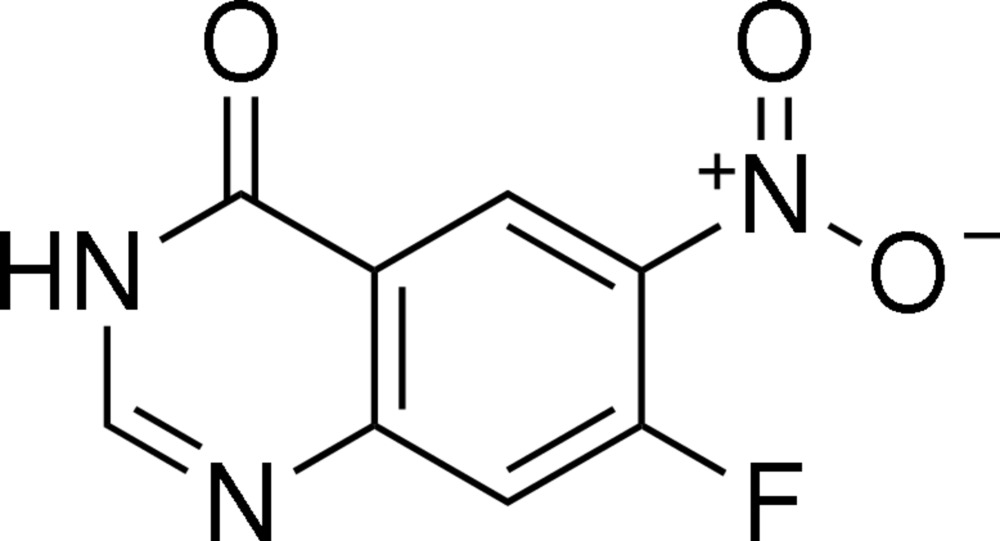


## Experimental

### 

#### Crystal data

C_8_H_4_FN_3_O_3_
*M* *_r_* = 209.14
Triclinic, 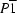

*a* = 5.6360 (11) Å
*b* = 8.409 (2) Å
*c* = 8.674 (2) Å
α = 79.38 (3)°
β = 89.23 (3)°
γ = 83.83 (3)°
*V* = 401.70 (16) Å^3^
*Z* = 2
Mo *K*α radiation
μ = 0.15 mm^−1^
*T* = 293 K
0.30 × 0.20 × 0.20 mm

#### Data collection

Enraf–Nonius CAD-4 diffractometer
Absorption correction: ψ scan (North *et al.*, 1968 ▶) *T*_min_ = 0.956, *T*_max_ = 0.971
1623 measured reflections
1461 independent reflections
1131 reflections with *I* > 2σ(*I*)
*R*_int_ = 0.018
3 standard reflections every 200 reflections intensity decay: 1%

#### Refinement

*R*[*F* ^2^ > 2σ(*F* ^2^)] = 0.050
*wR*(*F* ^2^) = 0.160
*S* = 1.00
1461 reflections
137 parameters
H-atom parameters constrained
Δρ_max_ = 0.23 e Å^−3^
Δρ_min_ = −0.25 e Å^−3^

### 

Data collection: *CAD-4 EXPRESS* (Enraf–Nonius, 1994 ▶); cell refinement: *CAD-4 EXPRESS*; data reduction: *XCAD4* (Harms & Wocadlo, 1995 ▶); program(s) used to solve structure: *SHELXS97* (Sheldrick, 2008 ▶); program(s) used to refine structure: *SHELXL97* (Sheldrick, 2008 ▶); molecular graphics: *SHELXTL* (Sheldrick, 2008 ▶); software used to prepare material for publication: *PLATON* (Spek, 2009 ▶).

## Supplementary Material

Crystal structure: contains datablocks global, I. DOI: 10.1107/S1600536809046984/pv2231sup1.cif

Structure factors: contains datablocks I. DOI: 10.1107/S1600536809046984/pv2231Isup2.hkl

Additional supplementary materials:  crystallographic information; 3D view; checkCIF report

## Figures and Tables

**Table 1:** Hydrogen-bond geometry (Å, °)

*D*—H⋯*A*	*D*—H	H⋯*A*	*D*⋯*A*	*D*—H⋯*A*
N1—H1*A*⋯O1^i^	0.86	1.98	2.815 (2)	165
C1—H1*B*⋯O2^ii^	0.93	2.47	3.396 (3)	179
C7—H7*A*⋯N2^iii^	0.93	2.50	3.422 (3)	171

Symmetry codes: (i) 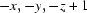; (ii) 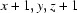; (iii) 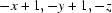.

**Fig. 1.**
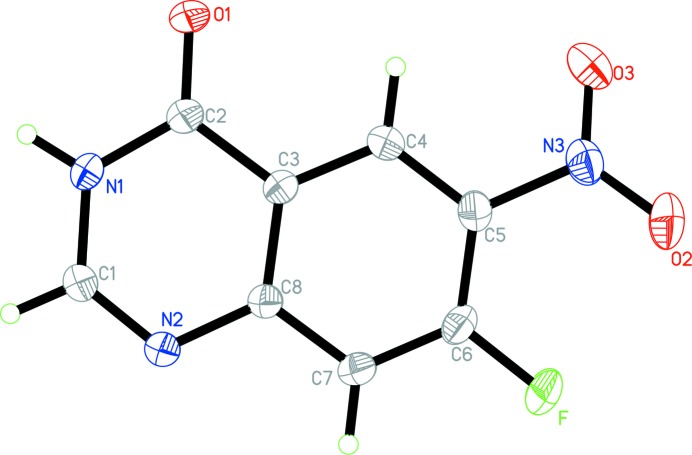
The molecular structure of the titlecompound with the atom numbering scheme. Displacement ellipsoids are drawn at 30% probability levels.

**Fig. 2.**
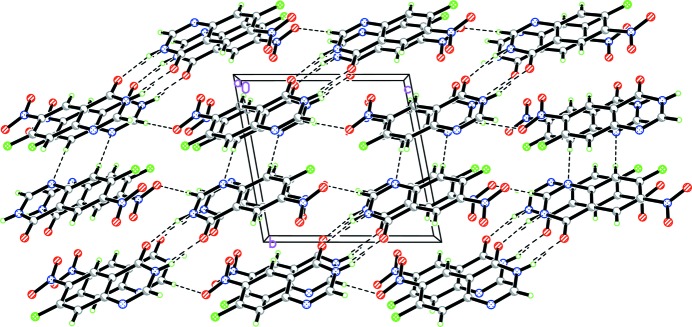
A packing diagram of the title compound. Hydron bonds are shown as dashed lines.

## supplementary crystallographic information

### Comment

4(3*H*)-Quinazolinone and its derivatives have been investigated extensively, owning to their important role in the synthesis of several multi-kinase inhibitors and to their potentially beneficial antitumor activities in many types of malignancies (Labuda *et al.*, 2009).

As part of our studies on the synthesis of 4(3*H*)-quinazolinone and its derivatives, the title compound, (I), which is used as the key intermediate (Rewcastle *et al.*, 1996), has been synthesized in our laboratory. We report herein the crystal structure of the title compound.

The molecule of the title compound is planar (Fig. 1). The quinazolinone moiety is essentially planar with maximum deviation for for any atoms being 0.0538 (14) for O1. The nitro group is twisted from the mean-plane of the quinazolinone ring by 12.0 (3)°. The bond lengths and angles in (I) are within normal ranges (Allen *et al.*, 1987). The crtstal structure of (I) is stabilized by classical and non-classical intermolecular hydrogen bonds of the types N—H···O, C—H···N and C—H···O; details have been provided in Table 1 and presented as a packing diagram in Fig. 2.

### Experimental

The title compound, was prepared by following a reported procedure (Rewcastle *et al.*, 1996). 7-Fluoroquinazolin-4(3*H*)-one (47.4 g, 0.29 mmol) was added to a mixture of concentrated H_2_SO_4_ (100 ml) and fuming HNO_3_ (100 ml), and heated at 373 K for 1 h. The crude product, 7-fluoro-6-nitroquinazolin-4(3*H*)-one, was obtained by pouring the reacting mixture onto ice-water (1500 ml). The crystals of (I) suitable for X-ray diffraction studies were obtained by recrystallization from acetic acid.

### Refinement

H atoms were positioned geometrically at distances N—H = 0.86 Å and C—H = 0.93 Å and constrained to ride on their parent atoms, with *U*_iso_(H) = 1.2 times *U*_eq_(parent atoms).

### Crystal data

**Table:**

C_8_H_4_FN_3_O_3_	*Z* = 2
*M**_r_* = 209.14	*F*(000) = 212
Triclinic, *P*1	*D*_x_ = 1.729 Mg m^−^^3^
Hall symbol: -P 1	Mo *K*α radiation, λ = 0.71073 Å
*a* = 5.6360 (11) Å	Cell parameters from 25 reflections
*b* = 8.409 (2) Å	θ = 9–13°
*c* = 8.674 (2) Å	µ = 0.15 mm^−^^1^
α = 79.38 (3)°	*T* = 293 K
β = 89.23 (3)°	Block, colorless
γ = 83.83 (3)°	0.30 × 0.20 × 0.20 mm
*V* = 401.70 (16) Å^3^	

### Data collection

**Table:**

Enraf–Nonius CAD-4 diffractometer	1131 reflections with *I* > 2σ(*I*)
Radiation source: fine-focus sealed tube	*R*_int_ = 0.018
graphite	θ_max_ = 25.3°, θ_min_ = 2.4°
ω/2θ scans	*h* = 0→6
Absorption correction: ψ scan (North *et al.*, 1968)	*k* = −10→10
*T*_min_ = 0.956, *T*_max_ = 0.971	*l* = −10→10
1623 measured reflections	3 standard reflections every 200 reflections
1461 independent reflections	intensity decay: 1%

### Refinement

**Table:**

Refinement on *F*^2^	Secondary atom site location: difference Fourier map
Least-squares matrix: full	Hydrogen site location: inferred from neighbouring sites
*R*[*F*^2^ > 2σ(*F*^2^)] = 0.050	H-atom parameters constrained
*wR*(*F*^2^) = 0.160	*w* = 1/[σ^2^(*F*_o_^2^) + (0.1*P*)^2^ + 0.12*P*] where *P* = (*F*_o_^2^ + 2*F*_c_^2^)/3
*S* = 1.00	(Δ/σ)_max_ < 0.001
1461 reflections	Δρ_max_ = 0.23 e Å^−^^3^
137 parameters	Δρ_min_ = −0.25 e Å^−^^3^
0 restraints	Extinction correction: *SHELXL97* (Sheldrick, 2008), Fc^*^=kFc[1+0.001xFc^2^λ^3^/sin(2θ)]^-1/4^
Primary atom site location: structure-invariant direct methods	Extinction coefficient: 0.062 (16)

### Special details

**Table:**

Geometry. All e.s.d.'s (except the e.s.d. in the dihedral angle between two l.s. planes) are estimated using the full covariance matrix. The cell e.s.d.'s are taken into account individually in the estimation of e.s.d.'s in distances, angles and torsion angles; correlations between e.s.d.'s in cell parameters are only used when they are defined by crystal symmetry. An approximate (isotropic) treatment of cell e.s.d.'s is used for estimating e.s.d.'s involving l.s. planes.
Refinement. Refinement of *F*^2^ against ALL reflections. The weighted *R*-factor *wR* and goodness of fit *S* are based on *F*^2^, conventional *R*-factors *R* are based on *F*, with *F* set to zero for negative *F*^2^. The threshold expression of *F*^2^ > σ(*F*^2^) is used only for calculating *R*-factors(gt) *etc*. and is not relevant to the choice of reflections for refinement. *R*-factors based on *F*^2^ are statistically about twice as large as those based on *F*, and *R*- factors based on ALL data will be even larger.

### Fractional atomic coordinates and isotropic or equivalent isotropic displacement parameters (Å^2^)

**Table:**

	*x*	*y*	*z*	*U*_iso_*/*U*_eq_	
F	0.1138 (3)	0.4301 (2)	−0.33786 (16)	0.0639 (5)	
N1	0.1677 (3)	0.1637 (2)	0.3773 (2)	0.0413 (5)	
H1A	0.1629	0.1209	0.4752	0.050*	
O1	−0.1399 (3)	0.0293 (2)	0.32253 (18)	0.0527 (6)	
C1	0.3360 (4)	0.2651 (3)	0.3291 (3)	0.0419 (6)	
H1B	0.4402	0.2828	0.4049	0.050*	
N2	0.3644 (3)	0.3390 (2)	0.1878 (2)	0.0406 (5)	
C2	0.0044 (4)	0.1261 (3)	0.2775 (2)	0.0380 (6)	
O2	−0.2795 (5)	0.3272 (3)	−0.3951 (2)	0.0932 (9)	
C3	0.0261 (4)	0.2086 (2)	0.1159 (2)	0.0331 (5)	
N3	−0.2740 (4)	0.2376 (3)	−0.2685 (2)	0.0486 (6)	
O3	−0.4036 (3)	0.1313 (2)	−0.2327 (2)	0.0630 (6)	
C4	−0.1305 (4)	0.1862 (3)	0.0011 (3)	0.0376 (6)	
H4A	−0.2523	0.1200	0.0276	0.045*	
C5	−0.1046 (4)	0.2618 (3)	−0.1509 (3)	0.0380 (5)	
C6	0.0818 (4)	0.3590 (3)	−0.1903 (2)	0.0396 (6)	
C7	0.2349 (4)	0.3832 (3)	−0.0789 (3)	0.0387 (6)	
H7A	0.3567	0.4491	−0.1068	0.046*	
C8	0.2091 (4)	0.3091 (2)	0.0771 (2)	0.0334 (5)	

### Atomic displacement parameters (Å^2^)

**Table:**

	*U*^11^	*U*^22^	*U*^33^	*U*^12^	*U*^13^	*U*^23^
F	0.0696 (10)	0.0883 (11)	0.0293 (8)	−0.0238 (8)	−0.0026 (6)	0.0104 (7)
N1	0.0493 (11)	0.0479 (11)	0.0260 (9)	−0.0160 (9)	−0.0022 (8)	0.0010 (8)
O1	0.0562 (11)	0.0618 (11)	0.0393 (9)	−0.0333 (8)	−0.0015 (7)	0.0086 (8)
C1	0.0434 (13)	0.0495 (13)	0.0342 (12)	−0.0154 (10)	−0.0056 (9)	−0.0051 (10)
N2	0.0413 (10)	0.0474 (11)	0.0338 (10)	−0.0172 (8)	−0.0030 (8)	−0.0019 (8)
C2	0.0401 (12)	0.0390 (12)	0.0338 (12)	−0.0107 (9)	−0.0005 (9)	−0.0001 (9)
O2	0.1106 (19)	0.123 (2)	0.0446 (12)	−0.0476 (15)	−0.0370 (12)	0.0113 (12)
C3	0.0356 (11)	0.0321 (11)	0.0306 (11)	−0.0060 (9)	−0.0001 (8)	−0.0019 (8)
N3	0.0487 (12)	0.0573 (13)	0.0421 (12)	−0.0065 (10)	−0.0087 (9)	−0.0138 (10)
O3	0.0533 (11)	0.0733 (13)	0.0676 (13)	−0.0208 (10)	−0.0126 (9)	−0.0172 (10)
C4	0.0372 (12)	0.0385 (12)	0.0385 (12)	−0.0115 (9)	−0.0015 (9)	−0.0063 (9)
C5	0.0404 (12)	0.0403 (12)	0.0333 (11)	−0.0028 (10)	−0.0060 (9)	−0.0069 (9)
C6	0.0450 (13)	0.0434 (12)	0.0273 (11)	−0.0041 (10)	0.0026 (9)	0.0009 (9)
C7	0.0374 (12)	0.0412 (12)	0.0360 (12)	−0.0111 (9)	0.0028 (9)	0.0003 (9)
C8	0.0316 (11)	0.0358 (11)	0.0322 (11)	−0.0067 (8)	−0.0004 (8)	−0.0032 (8)

### Geometric parameters (Å, °)

**Table:**

F—C6	1.328 (2)	C3—C4	1.391 (3)
N1—C1	1.354 (3)	C3—C8	1.401 (3)
N1—C2	1.371 (3)	N3—O3	1.208 (3)
N1—H1A	0.8600	N3—C5	1.462 (3)
O1—C2	1.222 (3)	C4—C5	1.367 (3)
C1—N2	1.284 (3)	C4—H4A	0.9300
C1—H1B	0.9300	C5—C6	1.401 (3)
N2—C8	1.381 (3)	C6—C7	1.361 (3)
C2—C3	1.455 (3)	C7—C8	1.394 (3)
O2—N3	1.211 (3)	C7—H7A	0.9300
			
C1—N1—C2	123.10 (18)	C5—C4—C3	119.7 (2)
C1—N1—H1A	118.4	C5—C4—H4A	120.1
C2—N1—H1A	118.4	C3—C4—H4A	120.1
N2—C1—N1	125.7 (2)	C4—C5—C6	119.8 (2)
N2—C1—H1B	117.2	C4—C5—N3	118.4 (2)
N1—C1—H1B	117.2	C6—C5—N3	121.8 (2)
C1—N2—C8	115.98 (18)	F—C6—C7	118.2 (2)
O1—C2—N1	121.96 (19)	F—C6—C5	120.7 (2)
O1—C2—C3	124.5 (2)	C7—C6—C5	121.1 (2)
N1—C2—C3	113.51 (19)	C6—C7—C8	120.0 (2)
C4—C3—C8	120.5 (2)	C6—C7—H7A	120.0
C4—C3—C2	120.43 (19)	C8—C7—H7A	120.0
C8—C3—C2	119.07 (19)	N2—C8—C7	118.51 (19)
O3—N3—O2	123.8 (2)	N2—C8—C3	122.58 (19)
O3—N3—C5	118.1 (2)	C7—C8—C3	118.91 (19)
O2—N3—C5	118.1 (2)		
			
C2—N1—C1—N2	0.6 (4)	O2—N3—C5—C6	−14.0 (4)
N1—C1—N2—C8	−0.8 (4)	C4—C5—C6—F	178.24 (19)
C1—N1—C2—O1	177.0 (2)	N3—C5—C6—F	−1.5 (4)
C1—N1—C2—C3	−1.4 (3)	C4—C5—C6—C7	−1.7 (4)
O1—C2—C3—C4	3.2 (4)	N3—C5—C6—C7	178.6 (2)
N1—C2—C3—C4	−178.5 (2)	F—C6—C7—C8	−179.23 (19)
O1—C2—C3—C8	−175.9 (2)	C5—C6—C7—C8	0.7 (4)
N1—C2—C3—C8	2.5 (3)	C1—N2—C8—C7	−178.6 (2)
C8—C3—C4—C5	0.5 (3)	C1—N2—C8—C3	2.0 (3)
C2—C3—C4—C5	−178.60 (19)	C6—C7—C8—N2	−178.6 (2)
C3—C4—C5—C6	1.1 (3)	C6—C7—C8—C3	0.8 (3)
C3—C4—C5—N3	−179.19 (19)	C4—C3—C8—N2	178.01 (19)
O3—N3—C5—C4	−12.3 (3)	C2—C3—C8—N2	−2.9 (3)
O2—N3—C5—C4	166.3 (2)	C4—C3—C8—C7	−1.4 (3)
O3—N3—C5—C6	167.4 (2)	C2—C3—C8—C7	177.65 (19)

### Hydrogen-bond geometry (Å, °)

**Table:**

*D*—H···*A*	*D*—H	H···*A*	*D*···*A*	*D*—H···*A*
N1—H1A···O1^i^	0.86	1.98	2.815 (2)	165
C1—H1B···O2^ii^	0.93	2.47	3.396 (3)	179
C7—H7A···N2^iii^	0.93	2.50	3.422 (3)	171

Symmetry codes: (i) −*x*, −*y*, −*z*+1; (ii) *x*+1, *y*, *z*+1; (iii) −*x*+1, −*y*+1, −*z*.
